# Spontaneous Urethral Laceration in a Patient Experiencing Acute Ulcerative Colitis Flare

**DOI:** 10.1155/2020/9285071

**Published:** 2020-02-05

**Authors:** Ryan Morris, John Barnard, Chad Morley

**Affiliations:** ^1^Texas A&M Health Science Center, USA; ^2^West Virginia University Health Science Center, USA

## Abstract

Spontaneous urethral laceration in the female without associated external trauma is an exceedingly rare phenomenon. Most cases are related to childbirth or the presence of a concomitant pelvic fracture or penetrating injury. Herein, we present a novel case of spontaneous urethral laceration in a female which happened to occur during an acute flare of her previously diagnosed ulcerative colitis. The diagnosis of spontaneous urethral laceration is rare, and underlying etiology is often uncertain.

## 1. Introduction

Urethral injuries are commonly secondary to well-defined traumatic events such as blunt trauma, penetrating injuries, or iatrogenic injuries including traumatic instrumentation [1]. The overall incidence of trauma-related pelvic injury is thought to be 5-10% when associated with a pelvic fracture making this type of injury not uncommon [2].

Ulcerative colitis is a chronic inflammatory condition affecting mainly the large colon from the rectum ascending proximally. The common symptoms and side effects of UC are well known and described in the medical literature including pain, diarrhea, hematochezia, and increased risk of colon cancer [3]. Of the known genitourinary manifestations of gastrointestinal disease, spontaneous urethral laceration has not been previously reported in conjunction with an acute UC flare [4].

## 2. Case Report

MA is a 49-year-old Caucasian female with a past medical history pertinent for ulcerative colitis who presented to the emergency department of our tertiary care, rural Level I trauma center for evaluation of periurethral pain and dysuria. She reported that her pain started 2 weeks prior to presentation while she was seated on the toilet straining to void. Due to symptoms of her UC, she had been experiencing frequent diarrhea. While attempting to void, she experienced a sudden onset of excruciating lower pelvic pain. Her pain persisted, and she was subsequently evaluated at an outside ED later that evening. She was diagnosed with a suspected ulcerative colitis flare given her history. Her dysuria was diagnosed as a urinary tract infection, and she was treated with empiric antibiotics. Over the next 3 days, despite adherence to antibiotic therapy, her symptoms persisted. She then presented to her primary care physician who agreed with the outside ED findings. She was additionally treated for hemorrhoids and advised to continue her antibiotic course. The following day, she had a new onset of gross hematuria for which she was again evaluated at an outside ED. Ultimately, she was admitted to the outside facility for 11 days before being discharged with a urology follow-up. After another 5 days elapsed, she was seen by her gynecologist who expressed concern for hematoma vs. abscess vs. malignancy due to an ulcerated, ecchymotic mass near the urethra. The patient was then advised to be evaluated at a tertiary care facility.

**Figure 1:**
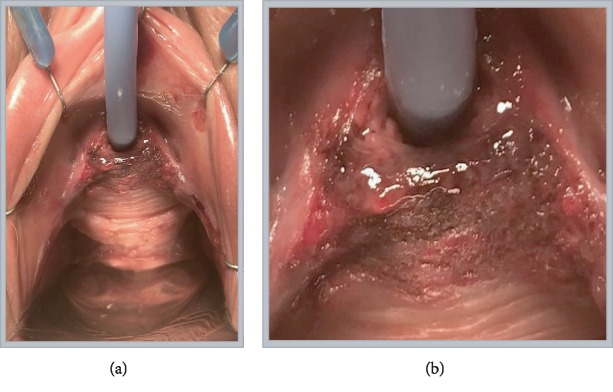
(a) Vaginal physical exam at time of initial cystoscopy. A Foley catheter can be seen entering the urethra and lone star retractor hooks. (b) A blown-up image showing the periurethral hematoma in greater detail.

On presentation to our facility, she was found to be tachycardic with HR of 116, normotensive, normothermic, and nontachypneic with oxygen saturations of 98%. She denied any fever, chills, nausea, vomiting, or other abdominal signs or symptoms. A review of her medical history revealed depression and ulcerative colitis but no previous urological history. Her previous surgical history included cholecystectomy and an OB GYN history of cesarean delivery. She was a lifetime nonsmoker and denies heavy alcohol use or any other drug use. She is allergic to Macrobid. On genitourinary exam, an ecchymotic lesion was present circumferentially around the distal urethra with ulceration inferiorly transitioning into the anterior vaginal vault (see Figure 1).

She reported that she had initially noticed this lesion shortly after her pain started 2 weeks prior to presenting at our facility. The rest of her physical examination was without abnormality. The initial impression was that this may be due to urethral prolapse, and the urology service was consulted. After attempted examination by urology in the ED, the decision was made to perform genitourinary exam under anesthesia and cystourethroscopy due to the patient's inability to tolerate physical examination at the bedside.

**Figure 2:**
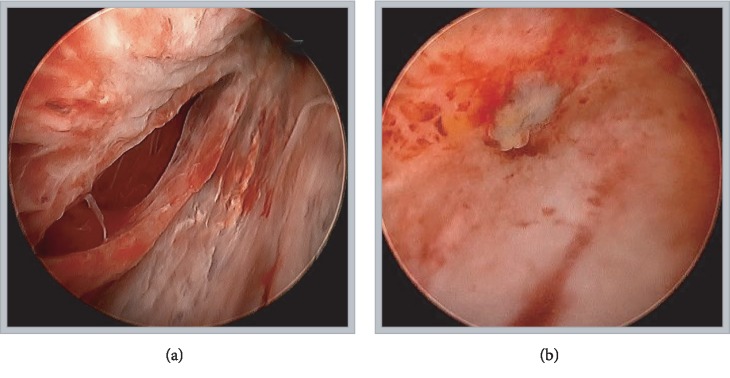
(a) Image of urethral laceration taken at time of initial cystoscopy. Full thickness tear through the urethra is visualized with periurethral soft tissue visualized through the defect. (b) Image of the right anterior lateral bladder wall, area of concern for potential colovesical fistula formation.

The examination under anesthesia revealed a firm indurated ecchymotic circumferential area around the urethra suspicious for hematoma with thrombosed urethral prolapse on the differential. Rigid cystourethroscopy was performed and showed a 1.0-1.5 cm longitudinal laceration of the urethra at the 7 o'clock position which would be the likely etiology of the hematoma (see Figure 2). Additionally, inspection of the bladder revealed numerous inflammatory lesions involving the trigone and bilateral lateral and anterior walls. Cold-cup biopsies were taken and sent to pathology, and the biopsy sites were fulgurated. Pathology later showed these lesions to be predominantly granulation tissue, blood clot, and inflammatory debris. There was also suspicion for colovesical fistula (see Figure 2) due do what appeared to be fecal material spilling into the bladder from some of the lesions. Hemostasis was achieved with spot coagulation, and an 18 Fr Foley catheter was placed at the end of the procedure which was maintained on discharge.

**Figure 3:**
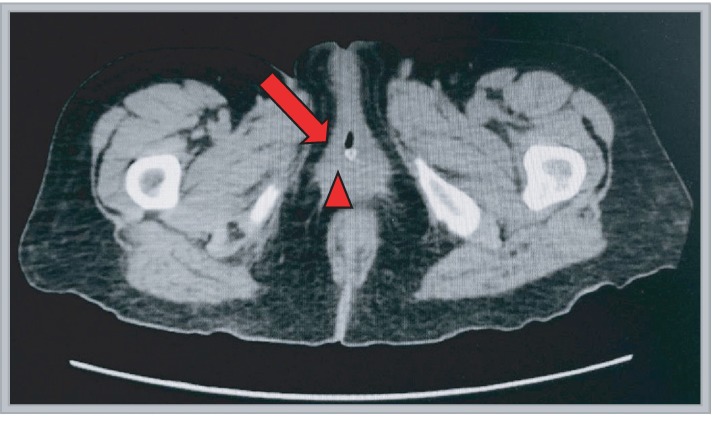
Axial cut CT image showing the urethral defect (*red arrow*) just anterior and slightly lateral to the Foley catheter (*arrowhead*) in place.

**Figure 4:**
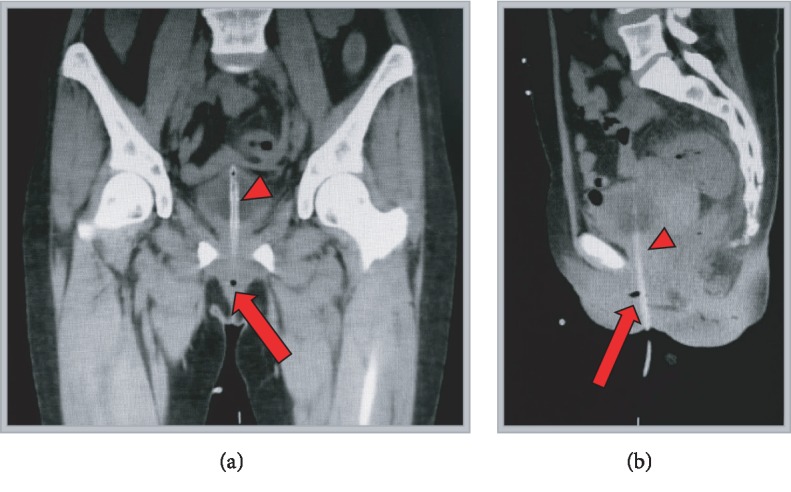
(a, b) Coronal and sagittal CT sections demonstrating the urethral defect (*red arrow*) near the in place Foley catheter (*red arrowhead*).

Her urine cultures from the operative intervention revealed Pseudomonas, and she was treated with culture-directed antibiotic therapy. A CT scan with and without contrast was ordered, and the patient was discharged with plans to follow up in the urology clinic in 2 weeks (see Figures 3 and 4).

She maintained her follow-up appointment and reported that she was doing well overall. She could not feel the hematoma like she could before, her pain was improving, and she denied any gross hematuria. She reported some drainage from around the catheter as well as some bladder spasms along with some mild fatigue. The physical exam confirmed significant improvement of her urethral and anterior vaginal wall hematoma. A repeat cystoscopy was scheduled for 2 weeks.

**Figure 5:**
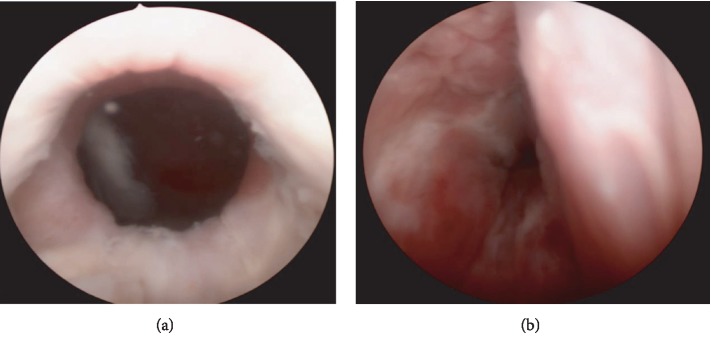
Urethra visualized on follow-up cystoscopy 4 weeks after initial cystoscopy. (a) Bladder neck visualized and without abnormality. (b) Distal urethra with healed laceration and resolution of hematoma.

No other acute events occurred before the 2-week follow-up. On repeat cystoscopic examination, her urethra was found to have healed well (see Figure 5), and there was no residual hematoma appreciated on cystoscopy or on physical exam; the bladder was unremarkable. The Foley catheter was removed, and the patient was discharged from urology care. She has continued to follow with the GI service for 6 months postpresentation and has experienced one relapse of her genitourinary symptoms found to be attributable to a drug-resistant Pseudomonas UTI.

## 3. Discussion

This patient's atraumatic urethral laceration without a clear inciting event other than prolonged straining to void is consistent with a spontaneous urethral laceration. Among the evidence supporting this conclusion are the urethral hematoma formation as blood is seen at the vaginal introitus in 80% of female urethral injuries [5], physical exam evidence collected by several different physicians in various specialties, and ultimately direct visualization of urethral laceration on cystoscopic evaluation. The role of her ulcerative colitis flare is more unclear. A definitive cause-effect relationship is difficult to ascertain. It is possible that increased tissue friability from robust inflammatory response predisposed the patient to urothelial injury. However, it was only after a prolonged episode of straining to void that her laceration occurred. The pathological evidence consistent with fibrosis and granulomatous tissue seen within the bladder walls indicates an ongoing, prolonged inflammatory response involving the urothelium.

The diagnosis of interstitial cystitis (IC) was considered given the known association with GU ulceration (Hunner's Ulcers). IC is typically associated with pelvic pain and pressure with the frequent urge to urinate in the absence of positive culture results; it is a diagnosis of exclusion. The patient had positive urine results during the initial hospitalization (Pseudomonas aeruginosa) and developed drug-resistant Pseudomonas UTI 3 months postpresentation. She has been followed for 6 months since presentation and has experienced control of her GU symptoms with treatment of her GI disease, with relapse during her recurrent Pseudomonal UTI. Also, she underwent flexible sigmoidoscopy which revealed erythematous and friable colonic mucosa, and the biopsy revealed granulation tissue and inflammatory debris.

Overall, the presence of a urethral laceration or complete disruption in a female without concomitant pelvic fracture is exceedingly rare. Literature review showed only 3 cases which were all associated with trauma despite the absence of a pelvic fracture with fall from standing causing a straddle injury and MVC being the underlying causes [6–8]. Our case is particularly unique due to the absence not only of a pelvic fracture but also of an underlying traumatic etiology. Review of the literature did not reveal any such case described previously. The concomitant UC flare has an unknown significance with respect to any predisposition to urothelial injury but should also be noted.

## 4. Conclusion

Spontaneous urethral lacerations in females, defined as a urethral laceration in the absence of a clear, traumatic inciting event, are exceedingly rare as they are typically the result of significant blunt trauma causing a pelvic fracture. Above, we present a unique case of female urethral laceration not only in the absence of pelvic fracture but also without any traumatic mechanism. Based on a review of the literature, such cases are extremely rare and we could not find previous reports. The patient was also experiencing an acute flare of ulcerative colitis, and any predisposition to urothelial injury from the resultant inflammatory state remains to be elucidated. The case highlights the need for awareness that urethral laceration is possible in the female population even in the absence of pelvic trauma.
